# Partner Choice and Context‐Dependent Sex Differences in Rat Rough‐and‐Tumble Play

**DOI:** 10.1111/ejn.70426

**Published:** 2026-02-10

**Authors:** Jackson R. Ham, Sergio M. Pellis, Robert J. McDonald

**Affiliations:** ^1^ Department of Neuroscience University of Lethbridge Lethbridge Canada

**Keywords:** individual variation, play fighting, rough‐and‐tumble play, sex differences, social behavior

## Abstract

Rough‐and‐tumble play (RTP) is the form of play most often observed in juvenile mammals. In rats, RTP typically consists of competing for access to the partner's nape, with both sex and social contexts influencing its frequency and structure. Although most studies employ dyadic tests, this design limits partner choice. Here, we investigated sex differences in partner preference and play frequency in Wistar rats using a group play paradigm. Juvenile rats were tested in mixed‐sex and same‐sex triads, allowing individuals to select partners. We measured playful nape attacks, defensive responses, and the distribution of play across partners. Results revealed that both sexes formed partner preferences, but only males exhibited sex‐based preferences, directing significantly more playful attacks toward females than males. Female rats showed no group‐level sex preference, though most individuals displayed consistent preferences for one partner, often, but not exclusively, for the male partner. Importantly, females initiated fewer nape attacks than males in mixed‐sex groups, but this sex difference disappeared in all‐female groups, where females initiated playful attacks at levels comparable to males. Thus, sex differences in RTP frequency were context dependent, emerging only when females were tested alongside males. These findings demonstrate two distinct forms of sex differences in the play of rats: partner choice and play frequency. Whereas males preferentially engage with females, females appear more flexible, forming idiosyncratic preferences independent of the partner's sex. Moreover, female play initiation is particularly sensitive to social context, highlighting the importance of group‐based testing for understanding naturalistic social decision‐making.

## Graphical abstract

When given partner choice in triadic play, male rats showed a robust preference for female partners, whereas females formed partner preferences that were not sex specific. Females initiated less play in mixed‐sex groups but played at male‐typical levels in all‐female groups, demonstrating that sex differences in rough‐and‐tumble play arise both from partner preference and from context‐dependent differences in play frequency.

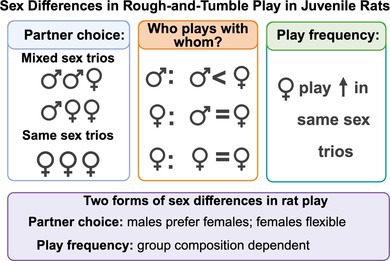

**Abbreviations**
PND: postnatal day
RTP: rough‐and‐tumble play

## 1 Introduction

As the name suggests, social play occurs between two or more individuals (Burghardt 2005; Pellis and Pellis 2009). One of the most common forms of social play is play fighting, or rough‐and‐tumble play (RTP), in which partners compete with one another to gain an advantage over one another, such as nuzzling, grooming, or biting a particular body target (Aldis 1975; Pellis et al. 2024). Whereas the target varies depending on the species, the targets competed over are often the same as those in serious fighting or those that are contacted during greeting, sex, or predation (Ham et al. 2023; Manitzas Hill, Ham, and Lilley 2022; Pellis 1988; Pellis et al. 2023). In addition to competition over body targets, an important facet of RTP is reciprocity. Indeed, individuals engaging in RTP cooperate to some degree so that both have the opportunity to attack and defend themselves (Ham, Pellis, and Pellis 2024; Palagi et al. 2016; Palagi 2023; Pellis and Pellis 2017).

Like many young mammals (Aldis 1975; Burghardt 2005; Fagen 1981; Pellis and Pellis 2009), juvenile rats, from weaning to sexual maturity, engage in RTP frequently (Meaney and Stewart 1981; Panksepp 1981; Pellis and Pellis 1990, 1997; Thor and Holloway 1984). RTP in rats mostly involves competing to access the partner's nape of the neck, which, if contacted, is gently nuzzled with the snout (Pellis and Pellis 1987; Siviy and Panksepp 1987). Because RTP in rats is easy to recognize and does not require training to induce (Pellis, Pellis, Ham, and Achterberg 2022), it has been used extensively to study the mechanisms regulating social behavior and the development of associated brain systems (e.g., Bagi et al. 2022; Ham, Szabo, et al. 2024; Marquardt et al. 2023; Reinhold et al. 2019; Stark et al. 2023; Veenema et al. 2013).

Rats show considerable individual variation in RTP, with some individuals being more playful than others (Achterberg et al. 2023; Ham and Pellis 2023; Lampe et al. 2019; Lesscher et al. 2021; Pellis and McKenna 1992). Given this variation, some rats might serve as more compatible play partners than others (Ham et al. 2025; Ham and Pellis 2023, 2024, 2025). Indeed, rats modify their behavior depending on the partner (Achterberg et al. 2023; Orsucci et al. 2025), for example, males modifying their play if partnered with females (Argue and McCarthy 2015). However, most studies of RTP employ the “dyadic test,” in which two animals are partnered. These partners are frequently of the same sex (Pellis, Pellis, Burke, et al. 2022; VanRyzin et al. 2020a). The dyadic test has been extremely useful in studying the RTP of rats but does have a limitation. In the wild, as juvenile rats age, they would not only have access to their littermates, but also peers from nearby litters, and therefore many potential partners of both sexes (Calhoun 1963; Schweinfurth 2020). Thus, in natural settings, rats would have choice with whom to play, a choice that laboratory rats tested in pairs are not afforded.

When given a choice, animals engaging in RTP typically prefer to play with partners of the same sex (e.g., *Saimiri sciureus* , Biben 1998; *Delphinapterus leucas* , Ham et al. 2023; *Macaca fuscata* , Shimada and Sueur 2018). However, these preferences for same‐sex play partners can change with age (Ham et al. 2022; Lilley et al. 2020) or depending on the type of RTP performed (Manitzas Hill, Ortiz, et al. 2022). Indeed, male Long‐Evans rats living and playing in their home cage under mixed‐sex conditions prefer to RTP with other males from postnatal day (PND) 26 to 35, but switch to preferring females from PND36 to 40 (Meaney and Stewart 1981). In contrast, females express no preference for sex until reaching sub‐adulthood, at around PND51 (Meaney and Stewart 1981). Similarly, when tested in pairs at the same age, Sprague–Dawley males play more with females than with males, whereas females play with male and female partners equally (Argue and McCarthy 2015).

In addition to sex differences in partner preferences, rats also show sex differences in the amount of RTP initiated. However, this varies from study to study, with some reporting that males play more (Argue and McCarthy 2015; Olioff and Stewart 1978) and others do not find significant differences between sexes (Orsucci et al. 2025; Stark et al. 2021).

Therefore, there are two forms of sex differences in rat RTP. There can be (1) sex differences in partner preference and (2) sex differences in the overall frequency of RTP. Given these two forms of sex differences, we first tested whether rats playing in mixed‐sex groups form partner preferences based on the sex of the partner. Using Wistar rats, a highly playful strain (S. M. Himmler et al. 2014), we grouped female and male rats with a male and female partner, testing the rats in triads in which the animals were unfamiliar with one another. By testing the rats in groups, the rats can choose to play with particular individuals while ignoring others, thus exhibiting preferences based on the number of playful attacks a rat directs toward members of the group (Ham et al. 2025; Ham and Pellis 2023, 2024, 2025). Given that males playing in pairs increase the frequency of RTP they engage in when with a female compared to when with a male, whereas females do not differ (Argue and McCarthy 2015), we predicted that males would prefer to play with females and ignore male partners but that females would not have a preference.

Second, given the influence of housing conditions on females, we tested whether the sex composition of the group influenced the frequency of play in female rats. As sex differences in the frequency of rat play are the strongest when rats are reared and/or tested in mixed‐sex groups (reviewed by S. M. Himmler et al. 2016), we predicted that females in mixed‐sex groups would play less than when in all‐female groups.

## 2 Materials and Methods

### 2.1 Subjects

Eight female and eight male Wistar rats were bred at the Canadian Centre for Behavioural Neuroscience after being purchased from Charles River (Kingston, NY). When the rats were around PND90, breeding pairs were formed. After 1 week, the males and females were separated from one another. The dams were housed in pairs until a few days before giving birth. When the pups were PND21, they were weaned and separated into same‐sex sibling pairs. A total of 16 female and 10 male rats were used for this study. All rats had food and water available *ad libitum*. The rats were housed on a 12‐h light–dark cycle (lights on between 0730 and 1930) in a room maintained at a constant temperature of 21°C–23°C. All care and testing procedures were approved by the University of Lethbridge Animal Welfare Committee (Protocols: 2307 and 2306, breeding and experimental protocols, respectively) in compliance with guidelines from the Canadian Council for Animal Care.

### 2.2 Apparatus

Play was tested in a large, clear Plexiglas enclosure (80 × 80 × 50 cm). The floor of the enclosure was covered in corncob bedding. Interactions were recorded with a digital video camera (Sony Handycam FDR‐AX53), which was placed over the enclosure at a 90° angle. Red lights were used to illuminate the enclosure.

### 2.3 Procedure

Rats were weighed and then moved to the test room where they were placed into the test enclosure with their cage mates for 2 consecutive days, spending 10 min of time in the enclosure each day to habituate them to the enclosure and room. This was done when the animals were PND28–29. Following the 2 days of habituation, the animals were weighed and then socially isolated for 2.5 h, with food and water provided *ad libitum*. All rats, including focal rats, were isolated for 2.5 h. After spending 2.5 h in isolation, the rats were moved to the testing room and placed into the enclosure for 20 min. The test schedule was repeated for 2 days (at PND30 and PND32). Habituation and testing occurred under red light. Each day, the animals were marked with a permanent marker pen (Sharpie) so they could be identified. Testing occurred between 0730 and 1200.

Groups were created that consisted of either (a) a focal female grouped with one female and one male, (b) a focal male grouped with one female and one male, or (c) a focal female grouped with two females. In all cases, the partners were unfamiliar (i.e., had never interacted with each other in the past) and unrelated, coming from different litters. For each trio condition (a–c), eight unique trios were formed. Because the primary aim was to assess how social context modulates female play initiation, the experimental design focused on group compositions necessary to isolate female behavior; an all‐male triad was therefore not included. Because of the limited cohort size (16 females, 10 males), animals participated in more than one test session across different group compositions. Rats could serve as focal animals in some sessions and as non‐focal partners in others, with test sessions separated in time. Critically, focal females were tested in both all‐female and mixed‐sex triads across separate sessions, allowing within‐subject comparisons of play initiation as a function of group composition. Group membership and focal status for each test session are detailed in Table S1. Sample sizes were based on group numbers used in our prior studies employing similar social play paradigms (Ham et al. 2025; Ham and Pellis 2023, 2024, 2025), rather than on an a priori power analysis, as variance estimates for triadic play measures are currently limited.

### 2.4 Behavioral Analysis

Following the play trials, the video files were analyzed using a combination of both normal speed and frame‐by‐frame analysis to score play behavior (B. T. Himmler et al. 2013; Pellis, Pellis, Burke, et al. 2022). All videos were scored noting the playful actions initiated by the focal animal. Playful attacks were scored when the snout of one rat was in contact with, or was directed toward, the nape of another rat (Pellis, Pellis, Burke, et al. 2022). Though rats can respond to playful attack in a variety of ways (Pellis, Pellis, Burke, et al. 2022), rotating along the horizontal access of the body, so that the attacked rat is in a supine position, is one of the most common responses and most frequently measured (e.g., Achterberg and Vanderschuren 2020; Siviy et al. 2023; Veenema et al. 2013). When rolling into a supine position, the rats are able to sustain close bodily contact (Stark et al. 2021). Rats less motivated to play are more inclined to evade an attack than roll over to supine (Pellis et al. 1997; Varlinskaya et al. 1999) as rolling to supine is thought to be an active solicitation of the partner, so facilitating the continuation of play (Achterberg and Vanderschuren 2023). Additionally, previous studies have found that depending on whether male rats are playing with a female or a male, the frequency of pinning is altered (Argue and McCarthy 2015). As such, we scored the total number of playful nape attacks, and whether the partner defended themselves by (a) rolling over into a supine “pinned” position or (b) with other tactics. The identity of the partner that was attacked was recorded so that the presence or absence of partner preferences could be determined.

We recognize the importance of blinding to experimental condition wherever possible. However, in the present study, this was not feasible because group membership could be readily inferred from the animals themselves. By PND28, rats exhibit clear sexual dimorphism, including visible external genitalia, making sex‐based group assignment immediately apparent during video scoring. As a result, scorers could not be blinded to group identity.

### 2.5 Statistical Analyses

Parametric tests were used after finding that all data were normally distributed with a Shapiro–Wilk test (*p* > 0.05). Paired *t*‐tests were used to analyze the frequency of nape attacks. The proportion of attacks that resulted in a defensive pin configuration was also tested with paired *t*‐tests.

In addition to the frequency of attacks, the proportion of attacks directed toward each partner was tested as there is a great deal of individual variation in the frequency of nape attacks launched (Achterberg et al. 2023; Ham and Pellis 2023). By testing both the frequency and proportion, we ensured that the “high players” do not bias the statistical tests. If both sexes were attacked equally, you would expect either sex to receive approximately 50% of the total nape attacks launched by the focal rat. As such, we used one‐sample *t*‐tests to assess whether one of the sexes was attacked significantly more than 50% of the time. Similarly, if the rats did not form preferences, both partners would be expected to receive approximately 50% of the total nape attacks launched by the focal subjects. We used one‐sample *t*‐tests to assess whether focal rats directed significantly more than 50% of their play toward either partner. The partner that received the greater proportion of attacks was designated as the “preferred partner,” regardless of sex or identity in the case of same‐sex groups.

Sex differences in the total frequency of play were tested with mixed‐effects analysis with a Bonferroni correction. To test partner differences in all‐female groups, the pairs were sorted based on frequency of nape attacks. This sorting was necessary as there was no other way to compare the partners given that there was no categorical difference between them (as there is in mixed‐sex groups). For each focal rat, we categorized one partner as preferred and the other as not preferred, with the preferred partner being attacked more than the not preferred partner. Though, until tested statistically, it was unclear whether “preferred” rats were played with more than “not preferred rats.”

All statistical tests and figures were performed and created using Prism Version 10 (GraphPad Software).

## 3 Results

### 3.1 Partner Preferences

#### 3.1.1 Female Focal Rats

**FIGURE 1:**
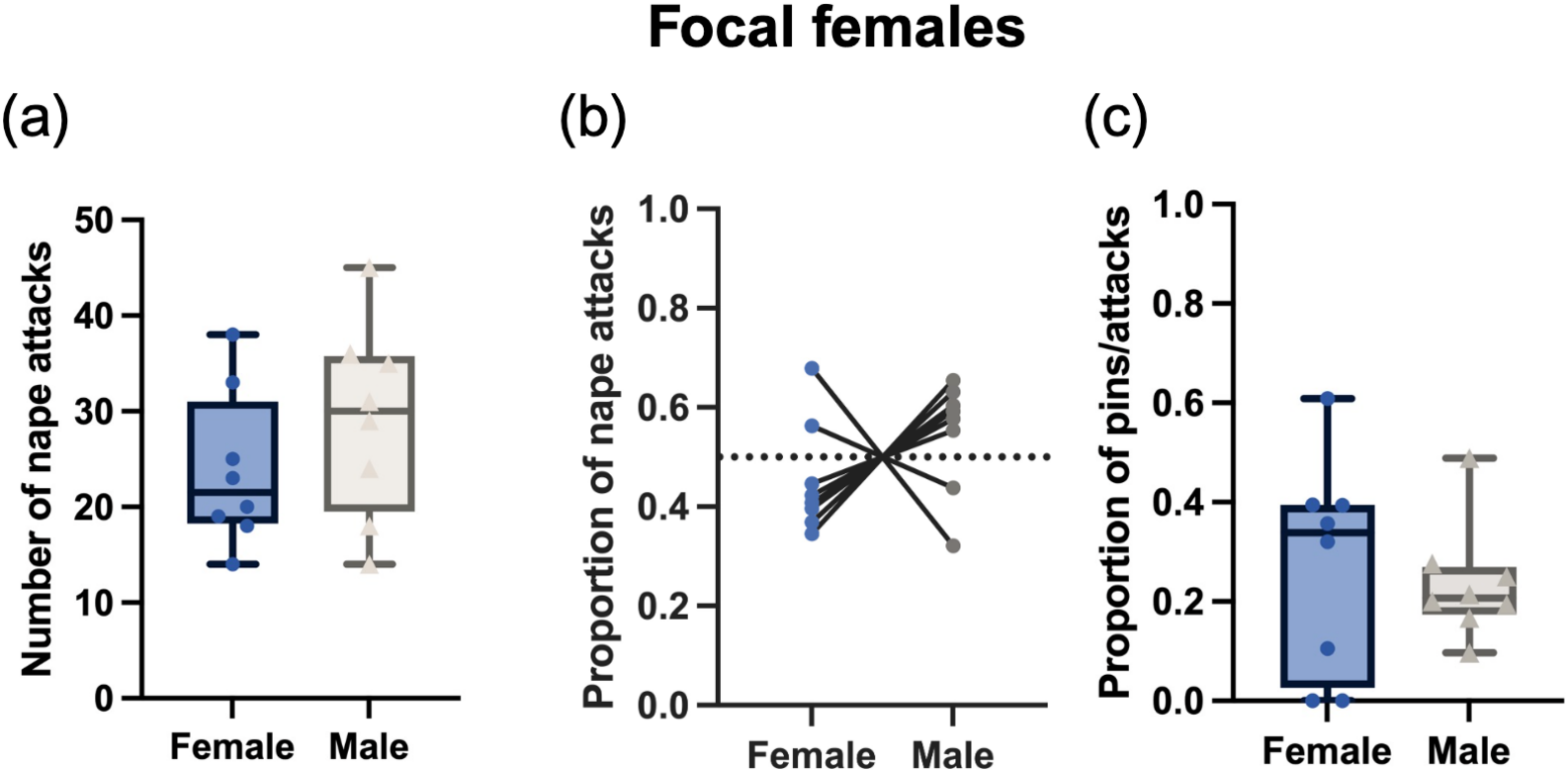
The number of nape attacks initiated by focal female rats playing in mixed‐sex groups that were directed toward either the male or female rat in their group is plotted (a). As there was a great deal of variation in the total number of nape attacks launched, the proportion of attacks was also plotted and determined that there was no preference for either sex (b). The proportion of nape attacks that resulted in a pin with either a male or female did not differ significantly (c).

The number of nape attacks female rats directed towards male and female partners in mixed‐sex groups (Figure 1a) did not differ significantly (paired *t*‐test: *t*_7_ = 1.249, *p* = 0.251). Similarly, the proportion of times females were attacked was not greater than 50%, suggesting that there was no difference in the proportion of attacks directed toward either sex (one‐sample *t*‐test: *t*_7_ = 1.171, *p* = 0.270) (Figure 1b). Despite not preferring a particular sex, female rats did show a significant preference for one of the partners playing with one partner significantly more than what you would expect by chance (one‐sample *t*‐test: *t*_7_ = 6.751, *p* = 0.0003). Most females preferred to play with males (*n* = 6); however, two preferred same‐sex partners (Figure 1b). When playfully attacked by a focal female, male and female partners were just as likely to end in a pin configuration (paired *t*‐test: *t*_7_ = 0.429, *p* = 0.681) (Figure 1c).

#### 3.1.2 Male Focal Rats

**FIGURE 2:**
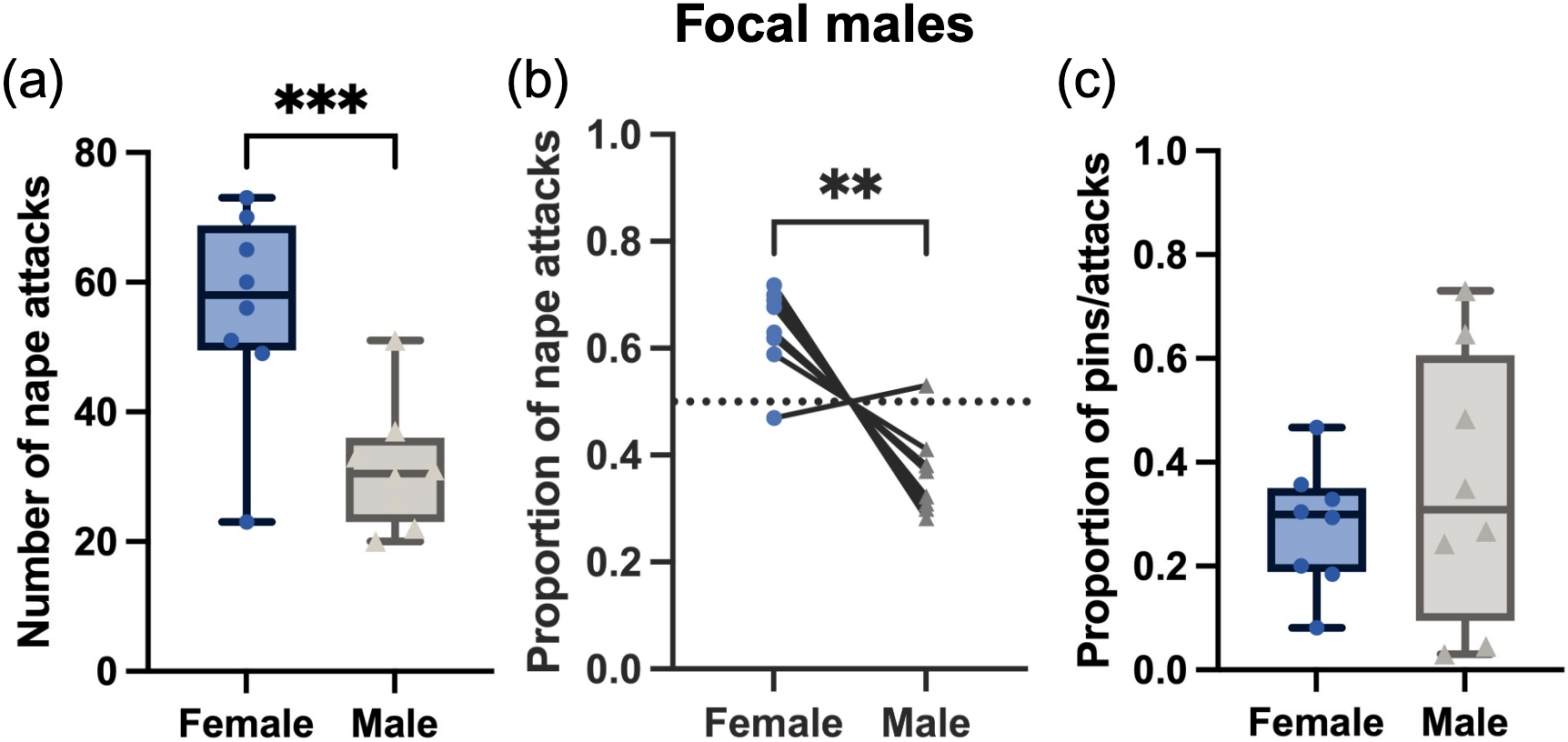
The number of nape attacks initiated by focal male rats and directed toward either the male or female rat in their group varied significantly indicating males preferred to play with females (a). As there was a great deal of variation in the total number of nape attacks launched, the proportion of attacks was also plotted, also indicating males preferred to play with females (b). The proportion of nape attacks that resulted in a pin with either a male or female did not differ significantly (c). **p < 0.01; ***p < 0.001.

The number of nape attacks male rats directed toward male and female partners in mixed‐sex groups (Figure 2a) differed significantly (paired *t*‐test: *t*_7_ = 5.444, *p* = 0.0010), with males attacking female partners more than males. Similarly, males directed a higher proportion of their play toward female partners than what would be expected by chance (one‐sample *t*‐test: *t*_7_ = 4.769, *p* = 0.0020) (Figure 2b). Seven of the eight males preferred to play with females over males. When playfully attacked by a male rat, male and female rats were just as likely to end in a pin configuration (paired *t*‐test: *t*_7_ = 0.7703, *p* = 0.466) (Figure 2c).

### 3.2 Context‐Dependent Sex Differences

**FIGURE 3:**
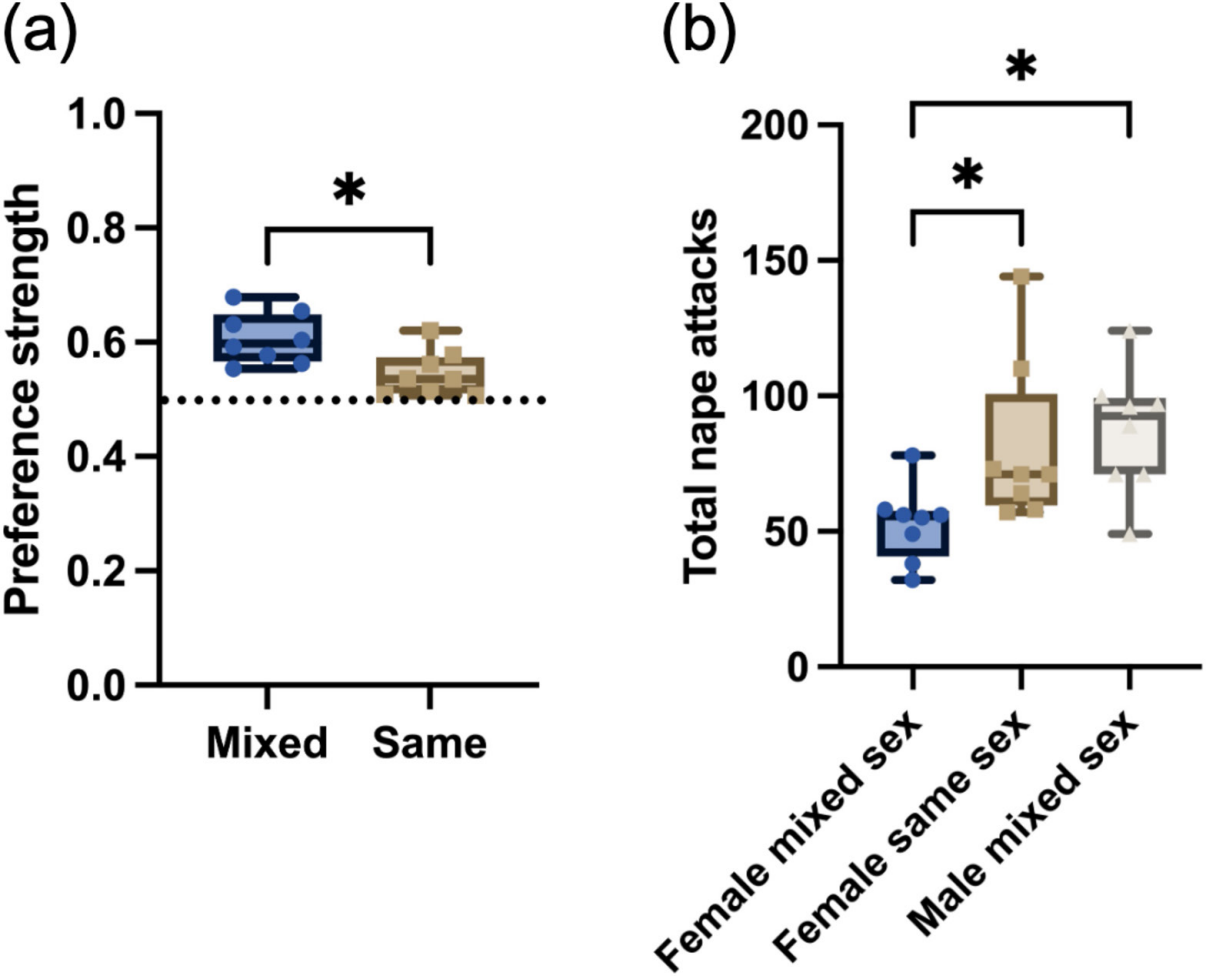
Preference strength for the preferred partner was weaker when focal females were playing in same‐sex groups compared to when playing in mixed‐sex groups (a). Focal males played significantly more than the females did when the total play directed at either a male or female partner, from the mixed‐sex test sessions, was summed (b). This difference, however, is not present when the total play males engaged in is compared to the total play the females engaged when playing in same‐sex groups or when the total play the focal females engaged in when playing in same‐sex groups is compared to when they played in mixed‐sex groups. *p < 0.05.

When playing in same‐sex groups, we found that female rats formed preferences for one of the two females, playing with one partner more than what you would expect by chance (one‐sample *t*‐test: *t*_7_ = 3.227, *p* = 0.015). However, when compared with the preference strength for the preferred partner in mixed‐sex groups (whether male or female), we found that female rats playing in all‐female groups had weaker partner preferences for the preferred partner in the mixed‐sex groups (paired *t*‐test: *t*_7_ = 3.273, *p* = 0.014). When playing in mixed‐sex groups, the focal female rats directed 61% to their preferred partner, whereas during same‐sex play, they directed 55% of their play toward their preferred partner (Figure 3a).

When the total number of nape attacks was compared (Figure 3b), we found males and females did not initiate the same amount of play (mixed‐effects model: *F*_1.871,13.09_ = 8.363, *p* = 0.0051). Post hoc comparisons revealed that the females in mixed‐sex groups played significantly less than when they were in all‐female groups (*p* = 0.031), whereas males played more than females in such groups (*p* = 0.016). This sex difference in play frequency, however, is not present for females playing in same‐sex groups compared to males playing in mixed‐sex groups (Figure 3b).

## 4 Discussion

Although sex differences are regularly reported in juvenile rat RTP (Argue and McCarthy 2015; Olioff and Stewart 1978; VanRyzin et al. 2019), many studies find no sex differences (Orsucci et al. 2025; Stark et al. 2021). Further complicating things, there are two forms of sex differences that can emerge in the RTP of rats. First, there can be sex differences in which partners are preferred (i.e., same‐sex or opposite sex partners). Second, there can be differences in the overall frequency of RTP. Here, we tested whether male and female rats prefer to play with same‐sex or opposite‐sex partners when given the choice (Argue and McCarthy 2015) and whether group composition influences how much female rats play. Our results demonstrate that whereas both females and males form partner preferences when playing in mixed‐sex groups, only males form preferences based on the sex of the partner. Indeed, for both females and males, the strength of partner preference was similar in mixed‐sex groups, but males played with female partners significantly more than with male partners. Although females formed preferences for one of the females in all‐female triads, this preference was significantly weaker than when playing in mixed‐sex groups. That is, whether they preferred to play with males or females in the mixed‐sex group, making a choice under mixed‐sex conditions is more polarizing than when playing in same‐sex groups. Females initiated significantly less playful attacks than males when playing in mixed‐sex triads; however, this sex difference disappeared when the females played in same‐sex triads. This suggests that the sex‐difference was due to the social setting and not because the females play less.

Our results, where males prefer females and females do not have a preference for either sex, reflect those of Meaney and Stewart (1981), despite differences in testing procedures. Here, we tested whether unfamiliar individuals would form preferences based on sex following a brief isolation period, whereas Meaney and Stewart (1981) measured play among familiar, related individuals in the home cage. Given that social isolation increases the motivation of rats to play (Panksepp and Beatty 1980; Pellis et al. 1997), this suggests that the males' preference to play with females is robust, not being masked by a general motivation to play. That is, the reward of playing itself did not outweigh playing with a preferred partner.

### 4.1 Sex Differences

Many species prefer to play with partners that are of the same sex (Biben 1998; Ham et al. 2023; Lilley et al. 2020; Shimada and Sueur 2018), including human children (Fry 2005). Here, we found that when given a choice, males initiated more playful attacks with females, whereas females showed no sex preference. Similar to when Sprague–Dawley rats are tested in pairs, we found that males initiated more playful attacks with females than males, whereas females play at similar levels regardless of the sex of the partner (Argue and McCarthy 2015).

Though sex differences are not always reported (Aguilar 2010; Field et al. 2006; S. M. Himmler et al. 2014; Madden and Zup 2014; Paul et al. 2014), we found that males launched more nape attacks than females when playing in mixed‐sex groups (Casto et al. 2003; Meaney and McEwen 1986; Pellis and Pellis 1990; Stockman and McCarthy 2017; Thor and Holloway 1982, 1984). This sex difference likely arises from early life hormone‐induced changes in the development of relevant brain mechanisms (see Pellis 2002 for a review). It is these underlying androgens during the neonatal period that seem to be responsible for the sex differences in levels of play initiation and do so by affecting the neural circuitry associated with play behavior (VanRyzin et al. 2020b). However, when the females played in same‐sex groups, we found that the sex difference was no longer present, with females playing just as much as their male counterparts, suggesting that social context is important in the expression of the sex difference in play. This suggests that whereas androgens may set a base frequency of play, other circulating hormones and neurotransmitters, such as endogenous opioids (Achterberg et al. 2019; Vanderschuren et al. 1995), can modulate how much rats play depending on the social context in which they find themselves (Achterberg et al. 2023).

The social context in which rats are reared seems to be particularly important, as sex differences are most apparent when the rats are reared in mixed‐sex groups (for a review, see S. M. Himmler et al. 2016). When reared in mixed‐sex groups, females typically play less than males, even when tested in same‐sex pairs. Interestingly, it seems that it is the female rats who are sensitive to mixed‐sex housing. Indeed, it is a reduction in female play, and not a change in the frequency of male play, that drives the sex effect (Achterberg et al. 2024; Argue and McCarthy 2015; Northcutt and Nwankwo 2018; Orsucci et al. 2025; Parent and Meaney 2008; Pellis et al. 1997; Siviy 2018; Siviy et al. 1990, 2003, 2017; Siviy et al. 2017; Siviy and Panksepp 1987; Thor and Holloway 1983, 1984; Veenema et al. 2013). Despite our rats being reared in same‐sex pairs after weaning, when tested in mixed‐sex groups, the sex differences in play frequency still emerged, suggesting that the influence of mixed‐sex groups has both a chronic and an acute effect on female play. That is, although chronic exposure to males in the home cage can decrease how much females play even when the females are tested in same‐sex pairs (e.g., Pellis and Pellis 1990), our present data show that males can also influence the play of females acutely, even for females housed in same‐sex pairs. Simply being exposed to males during a single play session decreases how much females play.

Alternatively, one factor contributing to reduced play initiation by focal females in mixed‐sex triads may be that males initiate play more frequently, thereby altering the opportunity for females to initiate play themselves. Because our analyses focused on play initiated by focal animals, we did not quantify initiation by non‐focal partners and therefore cannot distinguish changes in motivation from changes in opportunity. Importantly, however, females initiated play at male‐typical levels in all‐female triads, indicating that sex differences in play initiation are context dependent and emerge from social dynamics rather than fixed individual differences. Focal play initiation may also reflect reciprocal responses to partner behavior, an inherent feature of social interactions that further underscores the role of group context in shaping sex differences in play.

### 4.2 Individual Differences and Preference for Novelty

When tested in groups, where the individuals are of varying familiarity (i.e., cage mate, individual on the other side of a clear and perforated divider, or a complete stranger), males prefer to play with novel, unfamiliar partners over familiar cage mates (Ham and Pellis 2023). Although this has not yet been tested in females, one potential explanation for the preference for females we observed in males could be novelty. That is, the males, which are housed in same‐sex cages, find the opposite sex attractive due to their novelty even though both available partners are unfamiliar (i.e., the females are a novel sex and a novel partner, whereas the males are just a novel partner). In contrast, females might instead prefer partners that suit their individual idiosyncrasies and not those that are simply more novel.

Given that two of the eight females initiated more play with the same‐sex partner, it could be that females prefer to regulate aspects of the play experience, such as the degree of cooperation and competition in the play (Pellis et al. 2024) and/or the frequency of play (Achterberg et al. 2023). Interestingly, juvenile female rats seem to be more sensitive to the familiarity of their play partner, playing more with individuals that they are familiar with over those who are unfamiliar (Argue and McCarthy 2015). Additionally, sub‐adult female rats spend more time interacting with strains of greater genetic similarity, whereas males prefer strains that are more novel (Ippongi et al. 2025). Although the females in this study did not have a choice over individuals who are familiar versus those who are unfamiliar, this might explain why some choose to play with the more familiar, same‐sex partner.

One important caveat is the sample size of this study. Given that 6/8 females preferred the opposite sex, if we had a larger sample size, this likely would have been significant. Nonetheless, this still would not explain why some females prefer to play with males and why others prefer females. Likewise, one of the males preferred the same‐sex partner. Understanding why certain individuals deviated from the group trend could provide interesting insights into individual differences and how animals make social decisions when confronted with unfamiliar individuals.

## 5 Conclusion

Given that males play more when partnered with females than when with males, when playing in dyads, and that females play at similar levels regardless of the sex of the partner (Argue and McCarthy 2015), we used the “group play” paradigm to determine if these preferences hold true when the rats had a choice. Like Argue and McCarthy (2015), we found that males play more with females. Unlike the males, we found that females playing in groups did not prefer either sex at a group level, though females did form preferences for a given partner. Most preferred to play with the opposite sex, but some preferred to play with other females. Like previous reports, we found that males initiated more play than females, though this effect was only observed when females were playing in mixed‐sex groups. When playing in all‐female groups, females initiated just as many playful attacks as males playing in mixed‐sex groups. The results from this study suggest that males and females may decide with whom to play using different cues and that sex differences in playfulness only emerge under certain testing conditions.

## Author Contributions

**Jackson R. Ham:** conceptualization, data curation, formal analysis, funding acquisition, investigation, methodology, project administration, visualization, writing – original draft, writing – review and editing. **Sergio M. Pellis:** conceptualization, investigation, methodology, supervision, validation, writing – review and editing. **Robert J. McDonald:** conceptualization, data curation, formal analysis, funding acquisition, investigation, project administration, resources, supervision, validation, writing – review and editing.

## Funding

Funding was granted by the University of Lethbridge to Robert J. McDonald and Jackson R. Ham (University of Lethbridge Research Fund) and by the Natural Sciences and Engineering Research Council of Canada (NSERC) to Robert J. McDonald (RGPIN‐2020‐06929) and Jackson R. Ham (CGS‐D).

## Conflicts of Interest

The authors declare no conflicts of interest.

## Supporting information


**Table S1:** Composition of trios, including which day they were tested.

## Data Availability

The experimental data that support the findings of this study are available on Figshare at the following link: 10.6084/m9.figshare.30300142.
