# Supplementary material for: Partner Choice and Context‐Dependent Sex Differences in Rat Rough‐and‐Tumble Play
Source: Eur J Neurosci. 2026 Feb 10;63(3):e70426. doi: 10.1111/ejn.70426 (PMC12892013; doi:10.1111/ejn.70426)
Supplement: Supplementary file 1 — Table S1: Composition of trios, including which day they were tested. [file EJN-63-0-s001.docx]

**Supplementary Table 1.** Composition of trios, including which day they were tested.

| Test day | Focal ID # | Focal sex | Partner 1 ID # | Partner 1 sex | Partner 2 ID # | Partner 2 sex |
| --- | --- | --- | --- | --- | --- | --- |
| 1 | 90 | M | 74 | F | 88 | M |
| 1 | 91 | M | 75 | F | 89 | M |
| 1 | 92 | M | 76 | F | 95 | M |
| 1 | 86 | M | 94 | M | 77 | F |
| 3 | 94 | M | 92 | M | 70 | F |
| 3 | 95 | M | 91 | M | 71 | F |
| 3 | 89 | M | 90 | M | 72 | F |
| 3 | 93 | M | 73 | F | 88 | M |
| 1 | 70 | F | 79 | F | 82 | F |
| 1 | 71 | F | 78 | F | 83 | F |
| 1 | 72 | F | 81 | F | 85 | F |
| 1 | 73 | F | 80 | F | 84 | F |
| 2 | 70 | F | 78 | F | 90 | M |
| 2 | 71 | F | 79 | F | 89 | M |
| 2 | 72 | F | 80 | F | 94 | M |
| 2 | 73 | F | 81 | F | 91 | M |
| 2 | 74 | F | 82 | F | 93 | M |
| 2 | 75 | F | 83 | F | 92 | M |
| 2 | 76 | F | 84 | F | 87 | M |
| 2 | 77 | F | 85 | F | 88 | M |
| 3 | 74 | F | 83 | F | 79 | F |
| 3 | 75 | F | 82 | F | 78 | F |
| 3 | 76 | F | 85 | F | 81 | F |
| 3 | 77 | F | 84 | F | 80 | F |

M = male; F = female.
